# *Pediococcus acidilactici* Strain Alleviates Gluten-Induced Food Allergy and Regulates Gut Microbiota in Mice

**DOI:** 10.3389/fcimb.2022.845142

**Published:** 2022-04-22

**Authors:** Wenhui Fu, Chen Chen, Qiang Xie, Shimin Gu, Sha Tao, Wentong Xue

**Affiliations:** ^1^ College of Food Science and Nutritional Engineering, China Agricultural University, Beijing, China; ^2^ College of Information and Electrical Engineering, China Agricultural University, Beijing, China

**Keywords:** *Pediococcus acidilactici*, gluten, allergy, probiotic, microbiota

## Abstract

Wheat flour, the most important source of food globally, is also one of the most common causative agents of food allergy. Wheat gluten protein, which accounts for 80% of the total wheat protein, is a major determinant of important wheat-related disorders. In this study, the effects of *Pediococcus acidilactici XZ31* against gluten-induced allergy were investigated in a mouse model. The oral administration of *P. acidilactici XZ31* attenuated clinical and intestinal allergic responses in allergic mice. Further results showed that *P. acidilactici XZ31* regulated Th1/Th2 immune balance toward Th1 polarization, which subsequently induced a reduction in gluten-specific IgE production. We also found that *P. acidilactici XZ31* modulated gut microbiota homeostasis by balancing the *Firmicutes*/*Bacteroidetes* ratio and increasing bacterial diversity and the abundance of butyrate-producing bacteria. Specifically, the abundance of *Firmicutes* and *Erysipelotrichaceae* is positively correlated with concentrations of gluten-specific IgE and may act as a fecal biomarker for diagnosis. The evidence for the role of *P. acidilactici XZ31* in alleviating gluten-induced allergic responses sheds light on the application of *P. acidilactici XZ31* in treating wheat allergy.

## Introduction

Wheat flour is the most important source of food globally. However, in some predisposed individuals, wheat sensitivity conditions represented by wheat allergy, celiac diseases, and wheat intolerance can be triggered by different wheat proteins (Scherf, 2019). Wheat gluten proteins, which account for 80% of the total wheat proteins, are major determinants of important wheat-related disorders (Akagawa et al., 2007). The prevalence of wheat allergy is about 0.2–1.3%, and CD is known to affect approximately 1% of Europeans (Nwaru et al., 2014; Czaja-Bulsa and Bulsa, 2017). Wheat is the most common cause of severe allergic reactions in Chinese populations (Jiang et al., 2016).

Generally, food protein-induced allergic responses are primed in the intestinal mucosa, and the immunological response is accompanied by cytokine production, intestinal tissue lesions, and mucosal disorders (Nowak-Wegrzyn et al., 2017). In addition to host gastrointestinal cells, imbalances in intestinal microorganisms in patients with allergy disease have been widely reported, such as the reduction of *Lactobacillales*, *Bacteroidales*, and *Clostridiales* and changes in gut microbiota diversity (Goldberg et al., 2020; Joseph et al., 2021). Probiotics, in general, have therefore emerged as potential alternative therapeutics in the past decade. Current knowledge suggests that the oral administration of probiotics may contribute to antigen modification, repair of gut barrier function, restoration of gut microbiota, and systemic immune regulation (Nermes et al., 2013; Samak and Rao, 2013; Bunyavanich et al., 2016; Fu et al., 2019; Joseph et al., 2021). To date, the anti-allergic mechanism of probiotic treatment has not been fully elucidated, thus limiting further clinical applications. Different types of food allergies have been reported with different configurations of the gut microbiota. A decreased abundance of *Lactobacilli* and an increased abundance of *Staphylococcus aureus* seem to be associated with egg and milk allergies in children. In a shrimp tropomyosin-sensitized murine model, the population of *Bacteroidetes* decreased, but the population of *Firmicutes* increased (Fu et al., 2019; Mauras et al., 2019). Therefore, rigorous scientific efforts are still required to screen specific probiotics for different types of food allergy.

Sourdough is a mixture of flour, water, and fermentation strains represented by lactic acid bacteria and yeasts. Strains isolated from sourdough have shown promising results for hydrolyzing allergenic proteins in wheat, dairy, and soy (Scherf et al., 2016; Rui et al., 2019). The nature of the sourdough microbes, especially lactic acid bacteria associated with various peptidases, is considered one of the most important factors influencing food allergenicity (De Vuyst et al., 2009). We previously reported that *Pediococcus acidilactici XZ31* isolated from Chinese traditional sourdough showed a higher proteolytic activity on both casein and wheat protein substrates (Fu et al., 2020b). Recently, we further demonstrated that dough fermentation with *P. acidilactici XZ31* can promote the digestion of wheat proteins by pepsin and trypsin (Fu et al., 2021) and reduce the *in vitro* antigenic reaction (unpublished). Interestingly, *P. acidilactici* has been shown to attenuate constipation and balance the altered intestinal microbiota in BALB/c mice (Qiao et al., 2021). To date, it remains unclear if *P. acidilactici* is of functional importance against intestinal allergic responses.

Therefore, in the present study, we wish to assess the effect of *P. acidilactici XZ31* on alleviating gluten-induced food allergy and investigate the underlying mechanisms. We established a gluten-induced allergy mouse model and compared the allergenic symptoms treated with or without *P. acidilactici XZ31*. The underlying mechanism of the effect of *P. acidilactici XZ31* on the host was also evaluated, which laid a foundation for further clinical application.

## Materials and Methods

### Preparation of *P. acidilactici* XZ31

*P. acidilactici XZ31* was isolated from Chinese traditional sourdough as previously described (Fu et al., 2020b). *P. acidilactici XZ31* was cultured in fresh de Man, Rogosa, and Sharpe broth at 38°C for 24 h. The bacteria pellets were collected by centrifugation at 6,000 *g* for 10 min, freeze-dried, and then re-suspended in phosphate-buffered saline (PBS) for oral administration in the mouse model.

### Animals and Gluten Sensitization

All experiments were approved by the Beijing Municipal Science and Technology Commission of China. Female (3 weeks old) specific pathogen-free BALB/c mice were purchased from Sipeifu Biotechnology (Beijing, China) and housed in individual cages under controlled conditions of temperature (23 ± 3°C), humidity (50 ± 10%), and light (12/12-h light/dark cycle). The mice were maintained on AIN-93M diet (wheat-free) before and during sensitization.

**Figure 1:**
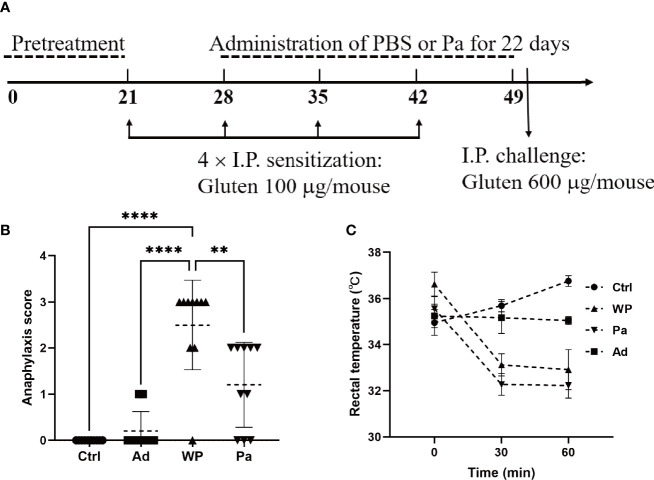
**(A)** Experimental design of *in vivo* immunization. **(B)** Assessment of clinical symptom after the last challenge. **(C)** Assessment of rectal temperature. Ctrl, non-immunized negative control; WP, group injected with gluten and adjuvant; Pa, treated with *Pediococcus acidilactici XZ31*; Ad, group injected with adjuvant. Data are shown as the mean value ± standard error of the mean of 10 independent experiments. The asterisk indicates a statistically significant difference (*P* < 0.05).

*In vivo* sensitization to wheat gluten was performed by using the protocol described by Caminero *et al*. with modifications (Caminero et al., 2019). The protocol is illustrated in **Figure 1A** and **Supplementary Table S1**. In brief, the mice were randomly divided into the following groups (*n* = 10): gluten-sensitized and challenged (WP), *P. acidilactici* XZ31-treated (Pa), non-immunized negative control (Ctrl), and group injected with adjuvant (Ad). Gluten from wheat (Shanghai Yuanye Bio-Technology) was prepared as described previously (Galipeau et al., 2011) with modifications. Briefly, gluten was dissolved in artificial gastric juice (Yuanye Co., Ltd., Shanghai, China; content: 0.2 N HCl, pH 1.5) for 2 h in 37°C water bath with pepsin (Sigma-Aldrich, 2,240 U/ml). Subsequently, the mixture was adjusted to pH 7.5 by the addition of 0.5 M NaHCO_3_. Trypsin (Sigma-Aldrich, 125 U/ml) was added, followed by shaking the mixture at 37°C for 1 h. Pepsin–trypsin (PT)-digested gluten was freeze-dried and stored at −20°C. The lypohilized samples were dissolved in PBS buffer (pH 7.0–7.2) before use, and the protein concentration was quantified by Bradford method (Akagawa et al., 2007).

Each mouse in the WP and Pa groups was sensitized by an intraperitoneal injection of 150 μl PT-digested gluten together with 50 μl aluminum adjuvant (Sigma-Aldrich). The total amounts of gluten were 100 μg on days 21, 28, 35, and 42, followed by a booster challenge with 600 μg gluten on day 50. The Ctrl group received 200 μl sterile PBS, and the Ad group received an equal amount of PBS containing 50 μl aluminum at each sensitization and challenge point. The Pa group was orally administered with a bacterial suspension (5 × 10 ^8^ to 6 × 10^8^ colony-forming units per mouse) once per day for 22 days from day 28 to 49, while the other groups received the same volume of sterile PBS. The mice were bled from the retro-orbital plexus on day 51. Fresh blood samples were incubated at room temperature for 30 min and centrifuged at 3,000 rpm for 10 min. The upper serum was taken and stored at -80°C. All mice were sacrificed on day 51, and organ and intestinal samples were collected for further analysis.

### Clinical Symptom Evaluation

The clinical symptoms of the mouse were observed within 60 min after an intraperitoneal challenge. The symptoms were evaluated using a standardized scoring system as detailed in **Table 1** (Fu et al., 2020a). Rectal temperature was measured before the gluten challenge and again at 30 and 60 min after the intraperitoneal challenge.

**Table 1:** Clinical anaphylaxis symptom scoring system of mice.

Score	Symptoms
0	No symptoms
1	Repetitive mouth/ear scratching and ear canal digging with hind legs
2	Decreased activity, self-isolation, and puffiness around the eyes and/or mouth
3	Periods of motionlessness for more than 1 min, lying prone on stomach, and decreased activity
4	No response to whisker stimuli and reduced or no response to probing
5	Tremor, convulsion, and death

### Measurement of Gluten-Specific Serum Antibody

Gluten-specific IgE and IgG2a in the serum were measured by enzyme-linked immunosorbent (ELISA) assay as previously described (Caminero et al., 2019). Briefly, 100 μl of diluted proteins (5 μg/ml in 50 mM carbonate–bicarbonate buffer, pH 9.6) was coated onto a microplate and incubated overnight at 4°C. The plates were blocked with bovine serum albumin (1% w/v in 0.02 M Tris-buffered saline) for 2 h at 37°C, followed by washing three times with Tris-buffered saline-Tween 20, and incubated for 2 hours with 100 μl of mouse serum (diluted to 1:5 for IgE measurement and 1:200 for IgG2a). After the washing cycles, the plates were incubated with HRP-conjugated goat anti-mouse IgE or IgG2a. The binding was assessed with a tetramethylbenzidine substrate kit. The results were measured at 450 nm using a microplate reader (Thermo Fisher Multiskan FC).

### Histological Analysis of Intestinal Tissue Sections

At sacrifice, the duodenum and colon were collected in 4% paraformaldehyde. The tissues were processed, paraffin-embedded, and stained with hematoxylin and eosin (H&E) for evaluation of tissue morphology. Mast cells and goblet cells were determined on paraffin-embedded sections after staining with toluidine blue and periodic acid–Schiff (PAS), respectively. Histological scoring was performed according to a scoring system described by Fu et al. (2018; 2020a) and by Ding and Wen (2018) with modifications (**Table 2**). The number of mast cells was quantified in at least five mice per group using ImageJ software.

**Table 2:** Mice intestinal histological scoring system.

Score	Tissue damage	
	Duodenum	Colon
0	No	No
1	Disorder of intestinal villus	Loss of goblets (slight)
2	Mucosal thickening	Loss of goblets (moderate)
3	Shedding of intestinal villus, loss of goblets (slight)	Loss of goblets (severe)
4	Shedding of intestinal villus, loss of goblets (moderate)	Absence of crypts, mucosal thickening (slight)
5	Shedding of intestinal villus, loss of goblets (severe)	Absence of crypts, extensive inflammatory infiltration (severe)

### Flow Cytometry

Spleen from the mouse was excised and kept in cold RPMI 1640 medium containing 10% fetal bovine serum. Single-cell suspensions were prepared and re-stimulated with Cell Stimulation Cocktail for 6 h (eBioscience, USA). T help (Th) cells were stained with FITC-conjugated anti-CD4, APC-conjugated anti-IFN-γ, and PE-conjugated anti-IL-4 (eBioscience, USA) according to the manufacturer’s instructions. The stained cell samples were detected using BD LSRII flow cytometry. FACS Diva8.0 software was used to analyze the data.

### Analysis of Intestinal Microbiota

Fecal samples were collected under sterile conditions and stored at −80°C until processing for DNA isolation. Total genome DNA from samples was extracted using the cationic detergent cetyl-trimethylammonium bromide method (Stewart and Via, 1993). DNA purity was monitored on 1% agarose gels (Sambrook, 2008). According to the concentration, DNA was diluted to 1 ng/μl using sterile water. 16S rRNA genes of distinct regions (16S V3 and V4) were amplified using specific primers with barcodes. The amplified products were mixed in equidensity ratios and purified with Qiagen Gel Extraction Kit (Qiagen, Germany). Sequencing libraries were generated using TruSeq^®^ DNA PCR-Free Sample Preparation Kit (Illumina, USA) following the manufacturer’s recommendations. The library concentrations and quality were checked using Qubit@ 2.0 Fluorometer (Thermo Scientific) and Agilent Bioanalyzer 2100 system, respectively. Lastly, the library was sequenced on Illumina NovaSeq platform. The original data was spliced and filtered out to get high-quality-tag sequences. The optimized sequences, with a limit of 3% distance, were gathered into operational taxonomic units (OTU) (Edgar, 2013), and the representative sequences were aligned against Silva database (http://www.arb-silva.de). Community richness and the diversity of each sample were determined by alpha diversity using the index of Chao and Shannon, which were calculated with QIIME (Version 1.7.0) and displayed with R software (version 2.15.3). Beta diversity analysis was calculated by QIIME software (version 1.9.1) and used as a comparative analysis of microbial communities in different samples.

### Statistical Analysis

Data are expressed as mean ± standard error of mean for each group. As noted, the statistical analysis was performed with Graph Prism using ANOVA test. *P <*0.05 was considered statistically significant.

## Results

### Clinical Symptom Observations of BALB/c Mice

We firstly examined the clinical allergenic response (**Figures 1B, C**). After the gluten intraperitoneal challenge, the WP group showed the highest anaphylaxis score and a persistent decrease of rectal temperature within 60 min, providing preliminary evidence of the validity of the model. The anaphylaxis score was significantly lower in the Pa group than in the WP group, and the rectal temperature recovered slightly after 60 min. The fluctuation of the rectal temperature was small in the Ctrl and Ad groups, and no obvious clinical reaction was observed.

### Morphological and Histological Observations of Intestinal Inflammation

**Figure 2:**
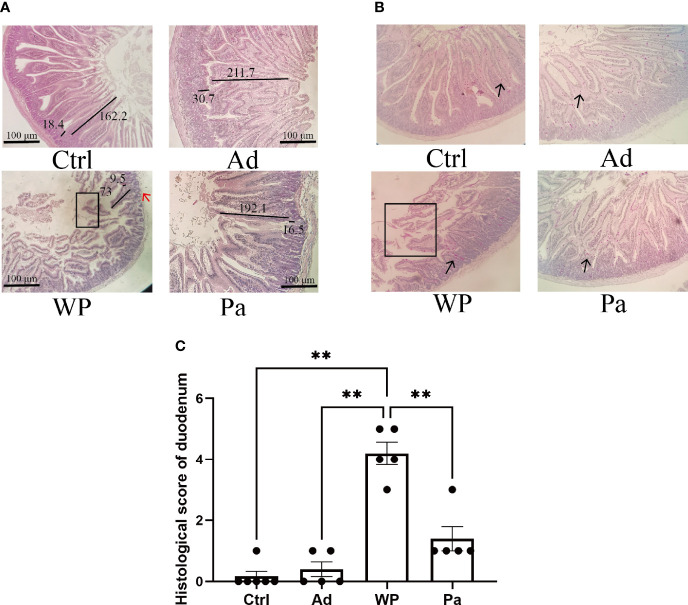
**(A)** Hematoxylin and eosin-stained duodenum in non-immunized negative control (Ctrl), group injected with adjuvant (Ad), group injected with gluten and adjuvant (WP), and treated with *Pediococcus acidilactici XZ31* (Pa). **(B)** Periodic acid–Schiff-stained duodenum in Ctrl, Ad, WP, and Pa. **(C)** Histological analysis of the duodenum. *n* = at least 5 mice per group. The results are presented as the mean value ± standard error of mean. The asterisk indicates a statistically significant difference (*P* < 0.05). The stained gut sections were photographed with a Leica microscope at ×100 magnification. The black box indicates shedding of the intestinal villus. The black line represents the villus height and crypt depth (μm). The red arrow indicates muscle damage. The black arrows indicate goblet cells (red pot).

**Figure 3:**
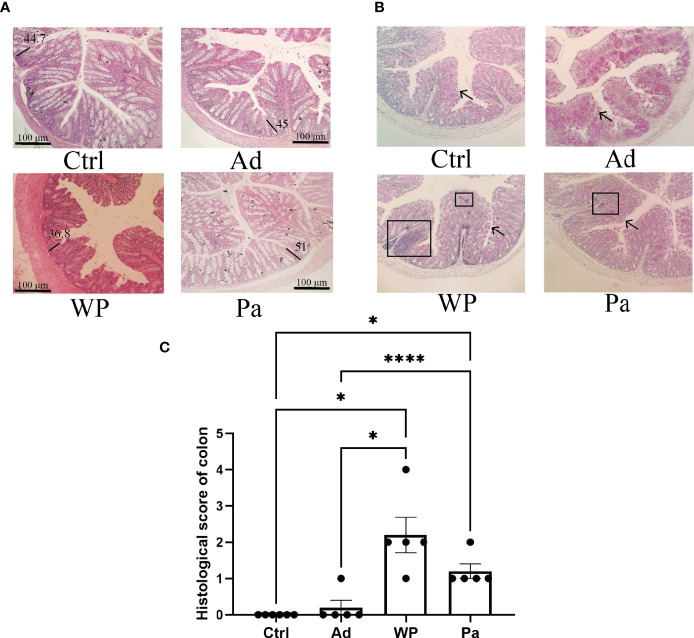
**(A)** Hematoxylin and eosin-stained colon in non-immunized negative control (Ctrl), group injected with adjuvant (Ad), group injected with gluten and adjuvant (WP), and treated with *Pediococcus acidilactici XZ31* (Pa). **(B)** Periodic acid–Schiff-stained colon in Ctrl, Ad, WP, and Pa. **(C)** Histological analysis of the colon. *n* = at least 5 mice per group. The results are presented as the mean value ± standard error of mean. The asterisk indicates a statistically significant difference (*P* < 0.05). The stained gut sections were photographed with a Leica microscope at ×100 magnification. The black line represents the crypt depth (μm). The black box indicates infiltration of inflammatory cells. The black arrows indicate goblet cells (red pot).

**Figure 4:**
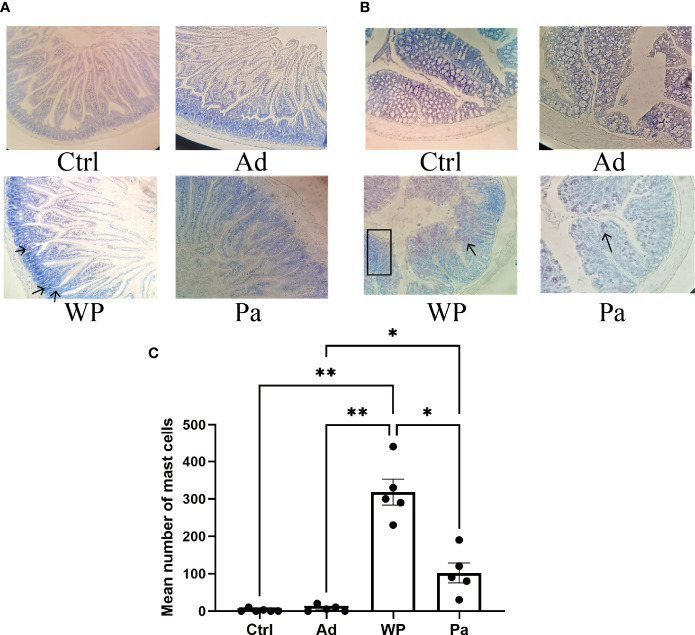
The mast cells in the duodenum **(A)** and colon **(B)** were stained with toluidine blue. The red arrow indicates infiltration of inflammatory cells. The total number of mast cells in the duodenum and colon is shown in **(C)**. *n* = at least 5 mice per group. Data are shown as the mean value ± standard error of mean. The asterisk indicates a statistically significant difference (*P* < 0.05). The stained gut sections were photographed with a Leica microscope at ×100 magnification. The black box indicates infiltration of inflammatory cells. The black arrows indicate mast cells (red pot).

Previous studies have shown that food allergy is always accompanied by gastrointestinal manifestations. We performed histopathological analyses of blinded tissue sections for the duodenum and colon from each group. H&E and PAS staining were performed to observe the intestinal morphology. As shown in **Table 2**, we examined the villus height, crypt depth, mucosal degeneration, and goblet cell distribution. The colonic infiltration of immune cells was also evaluated. Villus height and crypt depth correlated with the pathological score and demonstrated shedding of intestinal villi in tissues with greater pathological changes. Representative H&E staining results are shown in **Figures 2A**, **3A**. In the Ctrl and Ad groups, the duodenum showed a well-differentiated brush border structure. There was no significant difference in the histological score of the duodenum between the Ctrl group, Ad group, and Pa group (**Figure 2C**). In the WP group, gluten caused the duodenum villi to become shorter and disordered (**Figures 2A, B**, black box). Dysplastic glands invaded through the muscularis mucosa into the submucosa and tunica muscularis (**Figure 2A**, red arrow). Reduced crypt depth and extensive inflammatory infiltration were also observed in the colon of the mice in the WP group (**Figure 3B**, black box). In the Ctrl, Ad, and Pa groups, a large number of goblet cells were distributed evenly in the duodenum and colon (**Figures 2B**, **3B**, black arrow). The gut sections were also stained with toluidine blue for mast cells. As we can see in **Figures 4A, B**, a higher number of mast cells and extensive inflammatory cell infiltration (**Figure 4B**, black box) were observed mainly in the colon of the WP group. The inflammatory cell infiltration in the Pa group was less than that in the WP group. These results further demonstrated the role of *P. acidilactici XZ31* in alleviating gluten-induced allergic responses.

### Effect of *P. acidilactici* XZ31 Strain on T Cell Subtypes in Mice

**Figure 5:**
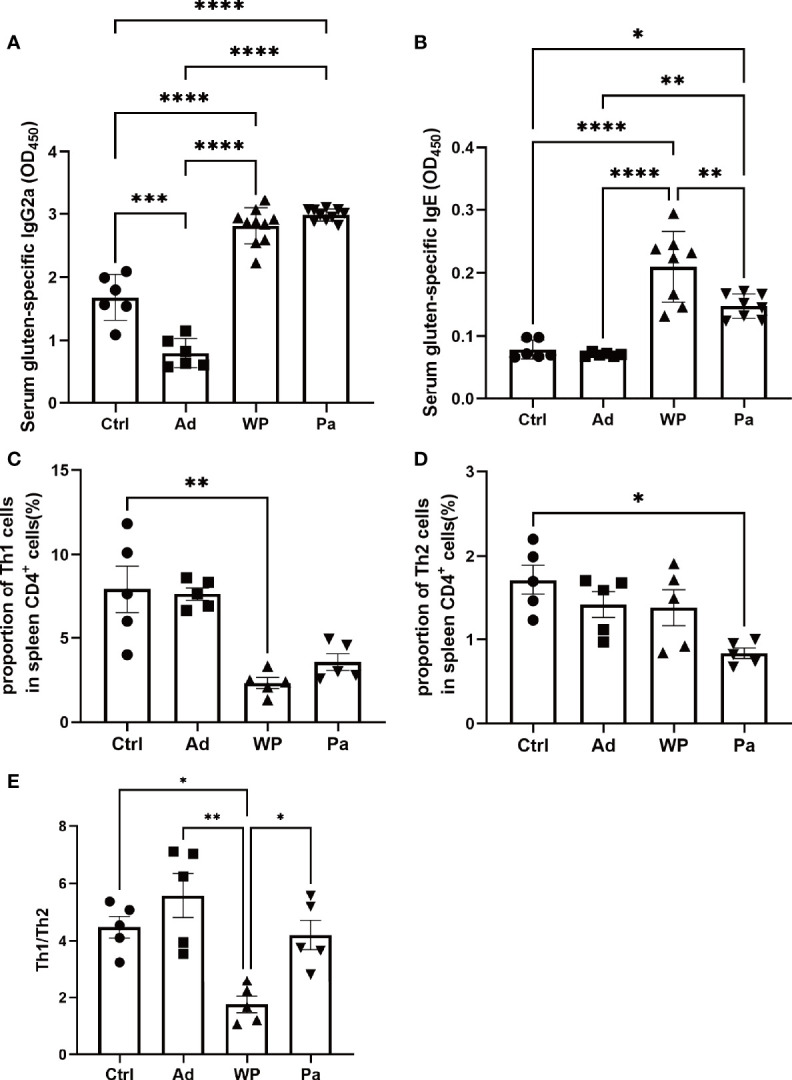
Serum gluten-specific IgG2a **(A)** and IgE **(B)** concentrations were measured by ELISA. The percentages of Th1 **(C)** and Th2 **(D)** in the spleens from different groups of mice were determined by FACS. **(E)** Th1/Th2 index. Data are shown as the mean value ± standard error of the mean (*n* ≥ 5). The asterisk indicates a statistically significant difference (*P* < 0.05).

To explore the mechanism of *P. acidilactici XZ31* in alleviating an allergenic reaction, we further characterize T cell differentiation in the spleen (anti-mouse IFN-γ for Th1 polarization and anti-mouse IL-4 for Th2 polarization). The IL-4 and IFN-γ production of splenic CD4 T cells is shown in **Supplementary Figure S1**. The production of IFN-γ was significantly lower in the WP group compared with that in the Ctrl group, and the production of IL-4 was higher and of IFN-γ was lower in the WP group than in the Pa group (**Figures 5C, D**). Food allergy is commonly a Th2-dependent disease and mediated by IgE, and the imbalance of Th1 and Th2 is an effective indicator of food allergy (Lee et al., 2018). As shown in **Figure 5E**, the Th1/Th2 ratio was significantly lower in the WP group. By contrast, we found that the ratio of Th1/Th2 increased significantly in the Pa group, indicating that the Th1/Th2 balance drifted to Th1 after *P. acidilactici XZ31* administration. As shown in **Figures 5A, B**, the levels of IgG2a and IgE were lower in the Ctrl and Ad groups. Gluten challenge resulted in a significant increase in gluten-specific IgE. The application of *P. acidilactici XZ31* significantly reduced the concentration of gluten-specific IgE. In contrast, the production of gluten-specific IgG2a was promoted by *P. acidilactici XZ31* compared to the WP group. These results show that *P. acidilactici XZ31* regulated the Th1/Th2 immune balance toward Th1 polarization, which subsequently induced a reduction in gluten-specific IgE production.

### Effect of *P. acidilactici* XZ31 Strain on the Homeostasis of the Gut Microbiota

**Figure 6:**
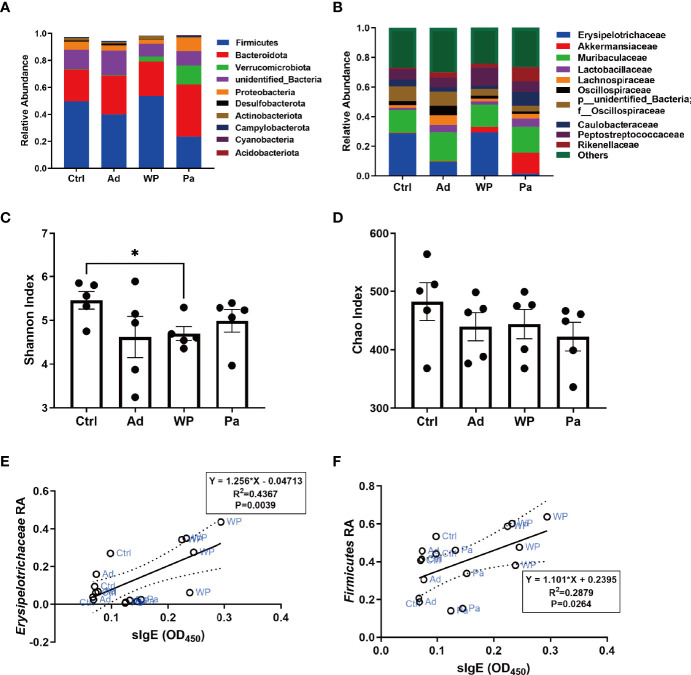
Bacterial composition of mouse gut at the phylum **(A)** and genus **(B)** levels. Significance test of Shannon index **(C)** and Chao index **(D)**. Correlation analysis of gluten-specific IgE concentration and abundance of two representative bacteria **(E**, **F)**. The dashed lines in **(E, F)** are the 95% confidence intervals of linear models (solid lines). RA, relative abundance. Data are shown as the mean value ± standard error of the mean of five independent experiments. The asterisk indicates a statistically significant difference (*P* < 0.05).

It is increasingly evident that intestinal bacteria are critical for regulating allergic responses to dietary antigens (Nermes et al., 2013; Iweala and Nagler, 2019). Gut microbial community composition was assessed to test the effect of the intervention of *P. acidilactici XZ31* on the intestinal microbiota homeostasis. The number of effective tags was more than 74% of the paired-end reads in all samples (**Supplementary Table S2**). The sequences that showed more than 97% similarity were assigned to the same OTU. The OTU coverage (≥0.999) indicated that a lower abundance of OTU has also been covered, which could accurately reveal the gut bacterial community structure of these samples (**Supplementary Figure S2**). At the phylum level (**Figure 6A**), *Bacteroidota* and *Firmicutes* are predominant in all groups. The proportion of *Bacteroidota* decreased slightly in the gluten sensitization group, while the proportion of *Firmicutes* increased compared with the Ad and Ctrl groups. The intake of *P. acidilactici XZ31* reduced the ratio of *Firmicutes*/*Bacteroidota* in the intestinal microbiota. At the family level (**Figure 6B**), the abundance of *Akkermansiaceae* and *Erysipelotrichaceae* was most affected by gluten and the *P. acidilactici XZ31* treatment. In short, the abundance of *Akkermansiaceae* and *Lactobacillaceae* in the Pa group was higher than that in the WP group. The increase in *Caulobacteraceae* and *Erysipelotrichaceae* after gluten sensitization was balanced by the administration of *P. acidilactici XZ31.* Additionally, the administration of *P. acidilactici XZ31* favored an increase of buryrate-producing bacteria, such as *Faecalibacterium prausnitzii* and *Collinsella aerofaciens* (**Supplementary Figures S3**, **S4**).

The bacterial community diversity and richness were assessed using the Shannon index and Chao index, respectively. The larger Shannon and Chao index values indicate higher diversity and richness of the sample. As shown in **Figures 6C, D**, there was no significant difference in community richness between the different groups. The *P. acidilactici XZ31* administration suppressed gluten-induced decline in intestinal microbiota diversity.

Linear regression analysis was performed to reveal the correlation between bacterial community in the gut and allergenic reaction. The results (**Figures 6E, F**) showed that the concentration of gluten-specific IgE antibody was positively related to the relative abundance of *Erysipelotrichaceae* and *Firmicute*s. Similar results were also found in Pearson analysis, of which the increase of gluten-specific IgE is accompanied by an increase in the relative abundance of *Erysipelotrichaceae* and *Firmicute*s (Pearson correlation analysis: *r* = 0.6608, *P* = 0.0039; *r* = 0.5365, *P* = 0.0264).

**Figure 7:**
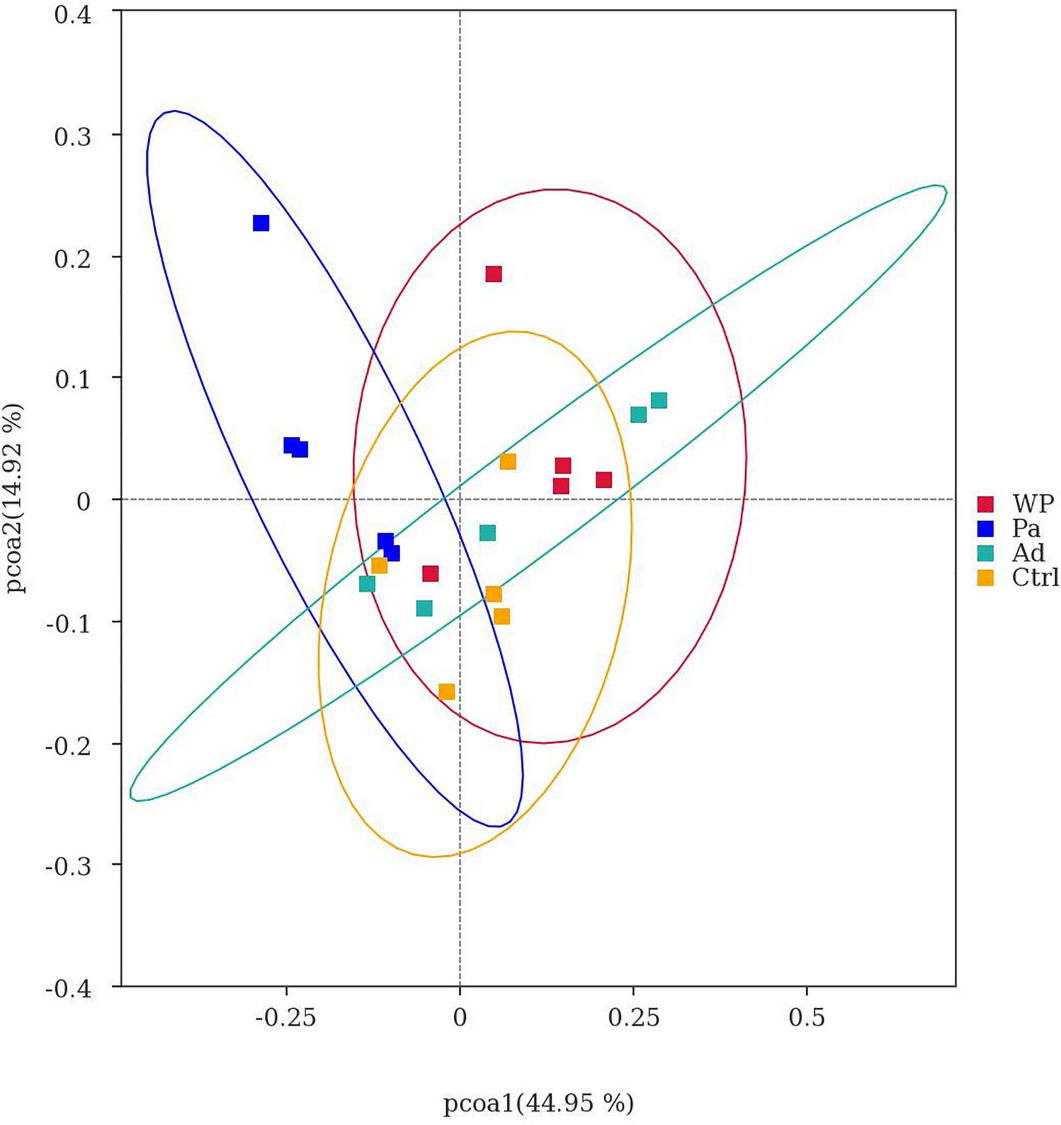
Principal co-ordinate analysis of different samples.

Beta diversity was used as a comparative analysis of microbial communities in different samples. Principal coordinate analysis (PCoA) revealed the correlation between gluten sensitization, Pa administration, and microbiota composition (**Figure 7**). Points spaced closer together are more similar in microbiota composition (Minchin, 1987). The WP group was far from the Pa group, implying a lower similarity in bacterial composition between them. Interestingly, the separation with the Ctrl and Ad groups increased in the Pa group compared to the WP group. The present results demonstrate that the administration of *P. acidilactici XZ3* can diminish gastrointestinal inflammation and allergic responses and increase the abundance of beneficial microbiota in the gut. We speculate that *P. acidilactici XZ3* may exhibit more beneficial effects in different types of intestinal homeostasis.

## Discussion

Previous studies have shown a promising outlook on the potential of probiotics, but the mechanism underlying the therapeutic or preventive effect of probiotics against food allergy remains unclear (Nermes et al., 2013; Pratap et al., 2020). In addition, the probiotic potential of specific strains varies, and there are currently no mixed or single culture for anti-allergic use. Thus, rigorous scientific efforts are still required to select specific strains with anti-allergenic potential and to reveal its potential mechanism for further clinical applications. We have previously reported that *P. acidilactici XZ31* fermentation degrades wheat toxic/immunogenic peptides and promote pepsin–trypsin digestion of wheat protein (Fu et al., 2021). Herein we studied the immunomodulatory potential of *P. acidilactici XZ31 in vivo* to further explore its probiotic characters and the underlying mechanisms. Toward this aim, a gluten-induced food allergy mouse model was established, and *P. acidilactici XZ31* was introduced. Because gluten is hard to dissolve and quantify in PBS buffers, we injected the mice with digested gluten. Unlike our study, Caminero *et al*. performed intragastric administration of the digested protein to the mice (Caminero et al., 2019). We believe that this would cause a secondary digestion of gluten and might affect its allergenicity. In addition, studies in mice have shown that injections are more effective than intragastric administration to establish a food allergy model (Chen et al., 2017). We found that the gluten challenge resulted in poor performance status and impaired intestinal homeostasis, indicating the successful establishment of an allergy mouse model.

The intestinal epithelial barrier serves as the dynamic interface between the host and the external environment and plays an important role in maintaining intestinal homeostasis (Moreno, 2006). Epithelium, mucus, and microbiota are required for the establishment of an intestinal barrier. Mucus secreted by goblet cells forms a physical barrier that impacts the adhesive interactions between intestinal epithelial cells and diverse antigens (Mowat and Agace, 2014). We found allergic mucosal disorders, such as reduced villus height and crypt depth, in the gluten sensitization mouse model. Loss of goblet cells is a potential cause of barrier dysfunction during gluten sensitization. Mast cells are considered as key effector cells in the elicitation of allergic symptoms. Previous reports have shown that the number of mast cells is closely related to the occurrence and development of intestinal inflammation (Feyerabend et al., 2011; Luo et al., 2019). Consistently, we also found an increase in the number of mast cells and inflammatory cell infiltration in the colon during gluten sensitization. Notably, oral administration with *P. acidilactici XZ31* attenuated the clinical allergic symptoms and intestinal injury in mouse.

The most common type of food allergy is an IgE-mediated reaction, which is a Th2-dependent disorder. Th2-cell cytokine-IL-4 is essential for IgE class switching, while Th1-cell cytokine IFN-γ is associated with IgG2a class switching. Thus, Th1/Th2 cytokine balance is crucial in food allergy, and a shift of Th1/Th2 immuno-dominance towards Th1 responses has been proposed as a strategy to correct the aberrant T cell immunity associated with food allergy (Coffman et al., 1989). The administration of *P. acidilactici XZ31* increased the proportion of Th1 cells in the spleen as well as reduced the concentration of gluten-specific IgE, suggesting that *P. acidilactici XZ31* can alleviate food allergy by suppressing the differentiation of allergic T cells.

In recent years, many studies have deciphered the relationship between probiotics and food allergy from the gut microbiota perspective. Probiotics alleviate food allergy by strengthening the epithelium and modulating the gut microbiota (Nermes et al., 2013; Pratap et al., 2020). However, the specific role of probiotics on the gut microbiota is controversial, as different types of food-induced allergies may show different gut microbiota disturbances. In an ovalbumin-sensitized mouse model, the administration of *Bifidobacterium infantis* attenuated ovalbumin-induced allergic mucosal disorders and increased the abundance of *Bacteroidetes*, *Firmicutes* (phylum level), *Lachnospiraceae S24-7*, *Rikenellaceae*, and *Ruminococcaceae* (family level) (Bo et al., 2017). Another study on the intervention of *B. infantis* on a shrimp tropomyosin-sensitized mouse model showed that the attenuation of the allergic reaction was accompanied by a decrease in the ratio of *Firmicutes*/*Bacteroidete* in the intestinal microbiota (Fu et al., 2017). Wheat allergy is among the most common food allergies, and the true prevalence of wheat allergy is often underestimated due to the lack of assessment methods. Currently, no major achievements have been made in understanding the role of probiotics on the wheat-induced imbalance of the mucosal ecosystem. In the present study, we found that the OUT enrichment in both healthy and allergic mice was largely taxa from *Firmicutes* and *Bcteroidota* phyla. *P. acidilactici XZ31* balanced the microbiota homeostasis by strengthening the *Bcteroidota*/*Firmicutes* ratio. *Firmicutes* and *Erysipelotrichaceae* were positively correlated with the concentrations of gluten-specific IgE and of particular interest for their potential as readily measurable fecal biomarkers for diagnosis. Additionally, oral administration with *P. acidilactici XZ31* not only modulated the properties of aberrant indigenous microbiota but also increased the abundance of beneficial bacteria, such as the genera *Alistipes* and *Akkermansiaceae*. Previous studies have shown the potential protective effect of *Alistipes* against colitis (Parker et al., 2020). *Akkermansiaceae* is known for its anti-inflammatory properties, including accelerating the development of intestinal epithelium and promoting T helper and T regulatory responses (Zhai et al., 2019; Ansaldo et al., 2021; Kim et al., 2021). Interestingly, buryrate-producing bacteria, including *Faecalibacterium prausnitzii* and *Collinsella aerofaciens* species, were increased by 79.5- and 32-fold, respectively, in the Pa group compared with the WP group (**Supplementary Figures S3**, **S4**). Butyrate is an energy source for the colonic epithelium. Besides this, butyrate was shown to induce the differentiation of Treg cells, thereby contributing to intestinal immune homeostasis (Arpaia et al., 2013). Of note is that *P. acidilactici XZ31* supplementation did not significantly increase its proportion but induced changes in the bacterial composition of gut microbiota. These results were in agreement with some previous studies showing an indirect function of probiotics in modulating the gut microbiota (Fu et al., 2017) but inconsistent with others suggesting that the role of probiotics is to compete successfully with other bacteria in the gut (Huang et al., 2017). The interaction between probiotics and intestinal microbiota is intriguing and deserves further investigation.

Taken together, the administration of *P. acidilactici XZ31* suppresses gluten-induced allergy possibly *via* the following ways: on the one hand, *P. acidilactici XZ31* attenuates gluten-specific IgE production by shifting the Th1/Th2 immune balance toward Th1 polarization; on the other hand, *P. acidilactici XZ31* alleviates intestinal inflammation by promoting the differentiation of goblet cells and increasing the abundance of beneficial intestinal bacteria. We previously reported the higher proteolytic activity on the wheat allergens of *P. acidilactici XZ31*. The evidence for the role of *P. acidilactici XZ31* in degrading wheat allergens *in vitro* and alleviating gluten-induced allergic responses *in vivo* shed light on the application of *P. acidilactici XZ31* in treating wheat allergy.

## Data Availability Statement

The datasets presented in this study can be found in online repositories. The name of the repository and accession number can be found below: NCBI, PRJNA795313.

## Ethics Statement

The Beijing Municipal Science and Technology Commission of China (number SYXK 2010-0036) approved all the animal studies described herein. The experiment was conducted according to the recommendations in the National Guide for the Care and Use of Laboratory Animals of China.

## Author Contributions

WF and WX conceptualized the study. WF, CC, QX, SG, ST, and WX curated the data and drafted the work. WF, CC, and WX provided overall supervision and coordinated the experimental activities. All authors contributed to the article and approved the submitted version.

## Funding

This project was supported by the National Natural Science Foundation of China (number 31872904) and the National Key Research and Development Program of China (number 2019YFC1605000).

## Conflict of Interest

The authors declare that the research was conducted in the absence of any commercial or financial relationships that could be construed as a potential conflict of interest.

## Publisher’s Note

All claims expressed in this article are solely those of the authors and do not necessarily represent those of their affiliated organizations, or those of the publisher, the editors and the reviewers. Any product that may be evaluated in this article, or claim that may be made by its manufacturer, is not guaranteed or endorsed by the publisher.

## References

[B1] Akagawa M. Handoyo T. Ishii T. Kumazawa S. Morita N. Suyama K. (2007). Proteomic Analysis of Wheat Flour Allergens. J. Agric. Food Chem. 55 (17), 6863–6870. doi: 10.1021/jf070843a 17655322

[B2] Ansaldo E. Farley T. K. Belkaid Y. (2021). Control of Immunity by the Microbiota. Annu. Rev. Immunol. 39 (1), 449–479. doi: 10.1146/annurev-immunol-093019-112348 33902310

[B3] Arpaia N. Campbell C. Fan X. Dikiy S. Veeken J. Deroos P.. (2013). Metabolites Produced by Commensal Bacteria Promote Peripheral Regulatory T-Cell Generation. Nature 504 (7480), 451–455. doi: 10.1038/nature12726 24226773 PMC3869884

[B4] Bo Y. Liang X. Sheng L. Liu X. Liu Z. (2017). Exploration of the Effect of Probiotics Supplementation on Intestinal Microbiota of Food Allergic Mice. Am. J. Transl. Res. 9 (2), 376–385. 28337267 PMC5340674

[B5] Bunyavanich S. Shen N. Grishin A. Wood R. Burks W. Dawson P.. (2016). Early-Life Gut Microbiome Composition and Milk Allergy Resolution. J. Allergy Clin. Immunol. 138 (4), 1122–1130. doi: 10.1016/j.jaci.2016.03.041 27292825 PMC5056801

[B6] Caminero A. McCarville J. L. Zevallos V. F. Pigrau M. Yu X. B. Jury J.. (2019). Lactobacilli Degrade Wheat Amylase Trypsin Inhibitors to Reduce Intestinal Dysfunction Induced by Immunogenic Wheat Proteins. Gastroenterology 156 (8), 2266–2280. doi: 10.1053/j.gastro.2019.02.028 30802444

[B7] Chen C. Lu L. H. Sun N. N. Li Y. Jia X. D.. (2017). Development of a Balb/C Mouse Model for Food Allergy: Comparison of Allergy-Related Responses to Peanut Agglutinin, Beta-Lactoglobulin and Potato Acid Phosphatase. Toxicol. Res. 6 (2), 251–261. doi: 10.1039/c6tx00371k PMC6061913 30090496

[B8] Coffman R. L. Savelkoul H. F. Lebman D. A. (1989). Cytokine Regulation of Immunoglobulin Isotype Switching and Expression. Semin. Immunol. 1 (1), 55. doi: 10.1016/0921-4488(91)90087-7 15630959

[B9] Czaja-Bulsa G. Bulsa M. (2017). What Do We Know Now About IgE-Mediated Wheat Allergy in Children? Nutrients 9 (1), 35. doi: 10.3390/nu9010035 PMC5295079 28054973

[B10] De Vuyst L. Vrancken G. Ravyts F. Rimaux T. Weckx S. (2009). Biodiversity, Ecological Determinants, and Metabolic Exploitation of Sourdough Microbiota. Food Microbiol. 26 (7), 666–675. doi: 10.1016/j.fm.2009.07.012 19747599

[B11] Ding A. Wen X. (2018). Dandelion Root Extract Protects NCM460 Colonic Cells and Relieves Experimental Mouse Colitis. J. Natural Medicines 72 (4), 857–866. doi: 10.1007/s11418-018-1217-7 29740735

[B12] Edgar R. C. (2013). UPARSE: Highly Accurate OTU Sequences From Microbial Amplicon Reads. Nat. Methods 10, 996. doi: 10.1038/nmeth.2604 23955772

[B13] Feyerabend T. B. Weiser A. Tietz A. Stassen M. Harris N. Kopf M.. (2011). Cre-Mediated Cell Ablation Contests Mast Cell Contribution in Models of Antibody- and T Cell-Mediated Autoimmunity. Immun. (Cambridge Mass.) 35 (5), 832–844. doi: 10.1016/j.immuni.2011.09.015 22101159

[B14] Fu L. Fu S. Wang C. Xie M. Wang Y. (2019). Yogurt-Sourced Probiotic Bacteria Alleviate Shrimp Tropomyosin-Induced Allergic Mucosal Disorders, Potentially Through Microbiota and Metabolism Modifications. Allergol. Int. 68 (4), 506–514. doi: 10.1016/j.alit.2019.05.013 31262632

[B15] Fu W. Liu C. Meng X. Tao S. Xue W. (2021). Co-Culture Fermentation of *Pediococcus Acidilactici XZ31* and Yeast for Enhanced Degradation of Wheat Allergens. Int. J. Food Microbiol. 347, 109190. doi: 10.1016/j.ijfoodmicro.2021.109190 33836445

[B16] Fu L. Song J. Wang C. Fu S. Wang Y. (2017). Bifidobacterium Infantis Potentially Alleviates Shrimp Tropomyosin-Induced Allergy by Tolerogenic Dendritic Cell-Dependent Induction of Regulatory T Cells and Alterations in Gut Microbiota. Front. Immunol. 8. doi: 10.3389/fimmu.2017.01536 PMC5686061 29176981

[B17] Fu L. Xie M. Wang C. Qian Y. Huang J. Sun Z.. (2020a). Lactobacillus Casei Zhang Alleviates Shrimp Tropomyosin-Induced Food Allergy by Switching Antibody Isotypes Through the NF-kappaB-Dependent Immune Tolerance. Mol. Nutr. Food Res. 64 (10), e1900496. doi: 10.1002/mnfr.201900496 32243079

[B18] Fu L. Xie M. Wang C. Wang H. Wang Y. (2018). Effects of Different Sensitization Pathways of Prawn Myosin on Sensitization of BALB/C Mice. Shi Pin Ke Xue 39, 10. doi: 10.7506/spkx1002-6630-201813025

[B19] Fu W. Xue W. Liu C. Tian Y. Zhang K. Zhu Z. (2020b). Screening of Lactic Acid Bacteria and Yeasts From Sourdough as Starter Cultures for Reduced Allergenicity Wheat Products. Foods 9 (6), 751. doi: 10.3390/foods9060751 PMC7353608 32517155

[B20] Galipeau H. J. Rulli N. E. Jury J. Huang X. Araya R. Murray J. A.. (2011). Sensitization to Gliadin Induces Moderate Enteropathy and Insulitis in Nonobese Diabetic-DQ8 Mice. J. Immunol. 187 (8), 4338–4346. doi: 10.4049/jimmunol.1100854 21911598 PMC3493154

[B21] Goldberg M. R. Mor H. Neriya D. M. Magzal F. Koren O. (2020). Microbial Signature in IgE-Mediated Food Allergies. Genome Med. 12 (1), 92. doi: 10.1186/s13073-020-00789-4 33109272 PMC7592384

[B22] Huang C.-H. Lin Y.-C. Jan T.-R. (2017). Lactobacillus Reuteri Induces Intestinal Immune Tolerance Against Food Allergy in Mice. J. Funct. foods 31, 44–51. doi: 10.1016/j.jff.2017.01.034

[B23] Iweala O. I. Nagler C. R. (2019). The Microbiome and Food Allergy. Annu. Rev. Immunol. 37 (1), 377–403. doi: 10.1146/annurev-immunol-042718-041621 31026410 PMC10445337

[B24] Jiang N. Yin J. Wen L. Li H. (2016). Retrospective Study of 1952 Severe Allergic Reactions in Chinese Population: Clinical Characteristics, Causes and Treatment. Zhonghua lin chuang mian yi he bian tai fan ying za zhi 10 (3), 247–254. doi: 10.3969/j.issn.1673-8705.2016.03.012

[B25] Joseph C. L. M. Sitarik A. R. Kim H. Huffnagle G. Fujimura K. Yong G. J. M.. (2021). Infant Gut Bacterial Community Composition and Food-Related Manifestation of Atopy in Early Childhood. Pediatr. Allergy Immunol. 33 (1), e13704. doi: 10.1111/pai.13704 34811824 PMC9301652

[B26] Kim S. Shin Y. C. Kim T. Y. Kim Y. Kweon M. N. (2021). Mucin Degrader Akkermansia Muciniphila Accelerates Intestinal Stem Cell-Mediated Epithelial Development. Gut Microbes 13 (1), 1–20. doi: 10.1080/19490976.2021.1892441 PMC7946046 33678130

[B27] Lee D. Kim H. S. Shin E. Do S. G. Lee C. K. Kim Y. M.. (2018). Polysaccharide Isolated From Aloe Vera Gel Suppresses Ovalbumin-Induced Food Allergy Through Inhibition of Th2 Immunity in Mice. Biomed. Pharmacother. 101 (1), 201–210. doi: 10.1016/j.biopha.2018.02.061 29494957

[B28] Luo Y. Meyer N. Jiao Q. Q. Scheffel J. Zimmermann C. Metz M.. (2019). Chymase-CreMcl-1fl/Fl Mice Exhibit Reduced Numbers of Mucosal Mast Cells. Front. Immunol. 10, 2399. doi: 10.3389/fimmu.2019.02399 31681290 PMC6803453

[B29] Mauras A. Wopereis H. Yeop I. Esber N. Delannoy J. Labellie C.. (2019). Gut Microbiota From Infant With Cow's Milk Allergy Promotes Clinical and Immune Features of Atopy in a Murine Model. Allergy 74 (9), 1790–1793. doi: 10.1111/all.13787 30887528 PMC6790679

[B30] Minchin P. R. (1987). An Evaluation of the Relative Robustness of Techniques for Ecological Ordination. VEGETATIO 69, 89–107. doi: 10.1007/BF00038690

[B31] Moreno F. J. (2006). Gastrointestinal Digestion of Food Allergens: Effect on Their Allergenicity. Biomed. Pharmacother. 61 (1), 50–60. doi: 10.1016/j.biopha.2006.10.005 17188456

[B32] Mowat A. M. Agace W. W. (2014). Regional Specialization Within the Intestinal Immune System. Nat. Rev. Immunol. 14 (10), 667–685. doi: 10.1038/nri3738 25234148

[B33] Nermes M. Salminen S. Isolauri E. (2013). Is There a Role for Probiotics in the Prevention or Treatment of Food Allergy? Curr. Allergy Asthma Rep. 13 (6), 622–630. doi: 10.1007/s11882-013-0381-9 23934549

[B34] Nowak-Wegrzyn A. Szajewska H. Lack G. (2017). Food Allergy and the Gut. Nat. Rev. Gastroenterol. Hepatol. 14 (4), 241–257. doi: 10.1038/nrgastro.2016.187 27999436

[B35] Nwaru B. I. Hickstein L. Panesar S. S. Roberts G. Muraro A. Sheikh A.. (2014). Prevalence of Common Food Allergies in Europe: A Systematic Review and Meta-Analysis. Allergy (Copenhagen) 69 (8), 992–1007. doi: 10.1111/all.12423 24816523

[B36] Parker B. J. Wearsch P. A. Veloo A. C. M. Rodriguez-Palacios A. (2020). The Genus Alistipes: Gut Bacteria With Emerging Implications to Inflammation, Cancer, and Mental Health. Front. Immunol. 11. doi: 10.3389/fimmu.2020.00906 PMC7296073 32582143

[B37] Pratap K. Taki A. C. Johnston E. B. Lopata A. L. Kamath S. D. (2020). A Comprehensive Review on Natural Bioactive Compounds and Probiotics as Potential Therapeutics in Food Allergy Treatment. Front. Immunol. 11. doi: 10.3389/fimmu.2020.00996 PMC7326084 32670266

[B38] Qiao Y. Qiu Z. Tian F. Yu L. Zhao J. Zhang H.. (2021). *Pediococcus Acidilactici* Strains Improve Constipation Symptoms and Regulate Intestinal Flora in Mice. Front. Cell Infect. Microbiol. 11. doi: 10.3389/fcimb.2021.655258 PMC8012752 33816357

[B39] Rui X. Huang J. Xing G. Zhang Q. Li W. Dong M. (2019). Changes in Soy Protein Immunoglobulin E Reactivity, Protein Degradation, and Conformation Through Fermentation With Lactobacillus Plantarum Strains. Lwt-Food Sci. Technol. 99, 156–165. doi: 10.1016/j.lwt.2018.09.034

[B40] Samak G. Rao R. K. (2013). Protection and Restitution of Gut Barrier by Probiotics: Nutritional and Clinical Implications. Curr. Nutr. Food Sci. 9 (2), 99–107. doi: 10.2174/1573401311309020004 24353483 PMC3864899

[B41] Sambrook J. (2008). Guidelines for Molecular Cloning Experiments (Beijing, China: Chemical Industrial Press), 308–313.

[B42] Scherf K. A. (2019). Immunoreactive Cereal Proteins in Wheat Allergy, non-Celiac Gluten/Wheat Sensitivity (NCGS) and Celiac Disease. Curr. Opin. Food Sci. 25, 35–41. doi: 10.1016/j.cofs.2019.02.003

[B43] Scherf K. A. Koehler P. Wieser H. (2016). Gluten and Wheat Sensitivities – An Overview. J. Cereal Sci. 67, 2–11. doi: 10.1016/j.jcs.2015.07.008

[B44] Stewart C. Via L. E. (1993). A Rapid CTAB DNA Isolation Technique Useful for RAPD Fingerprinting and Other PCR Applications. Biotechniques 14, 748–750. 8512694

[B45] Zhai R. Xue X. Zhang L. Yang X. Zhang C. (2019). Strain-Specific Anti-Inflammatory Properties of Two Akkermansia Muciniphila Strains on Chronic Colitis in Mice. Front. Cell. Infect. Microbiol. 9. doi: 10.3389/fcimb.2019.00239 PMC6624636 31334133

