# Supplementary material for: Pediococcus acidilactici Strain Alleviates Gluten-Induced Food Allergy and Regulates Gut Microbiota in Mice
Source: Front Cell Infect Microbiol. 2022 Apr 22;12:845142. doi: 10.3389/fcimb.2022.845142 (PMC9072736; doi:10.3389/fcimb.2022.845142)
Supplement: Supplementary file 1 [file DataSheet_1.docx]

**Figure S1**


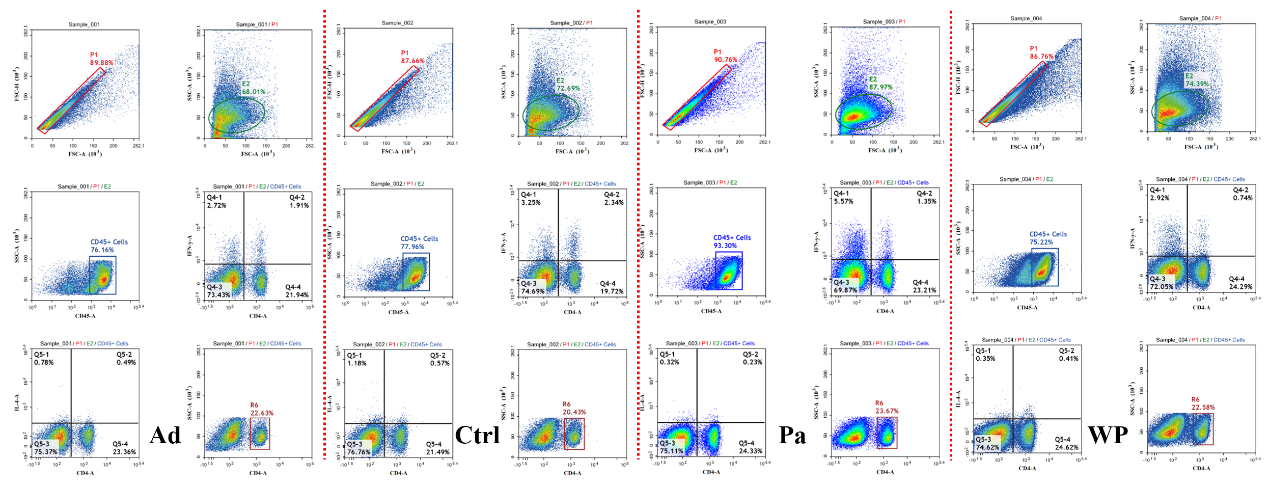


**Fig. S1.** Representative dot plots of flow cytometry.

**Figure S2**


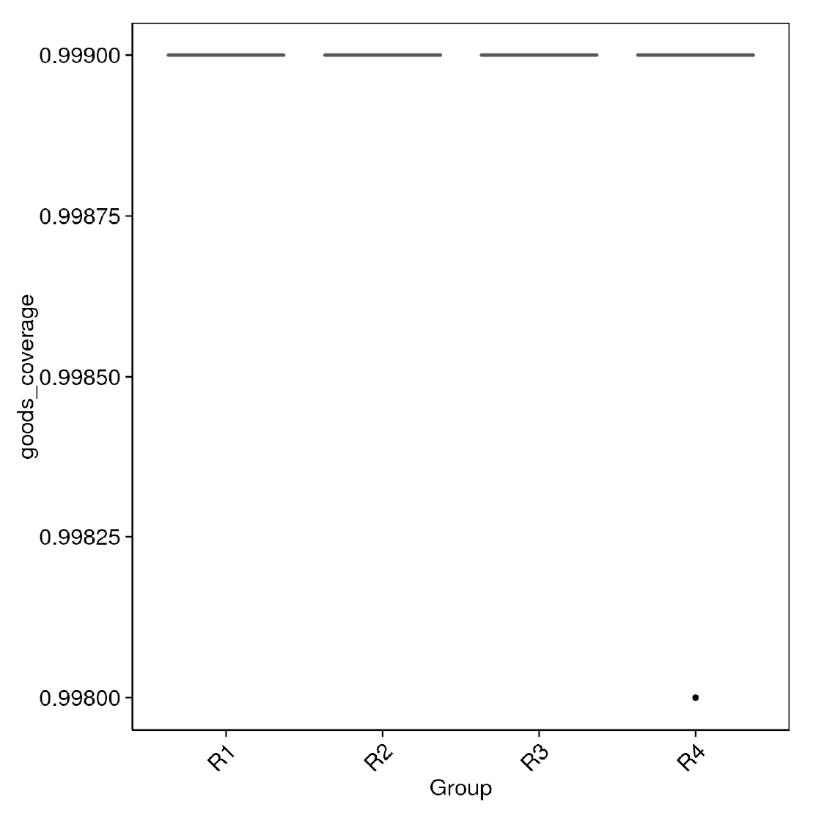


**Fig. S2.** OTU coverage in these samples. OTU, operational taxonomic unit; R1, Ctrl; R2, Ad; R3, WP; R4, Pa.

**Figure S3**


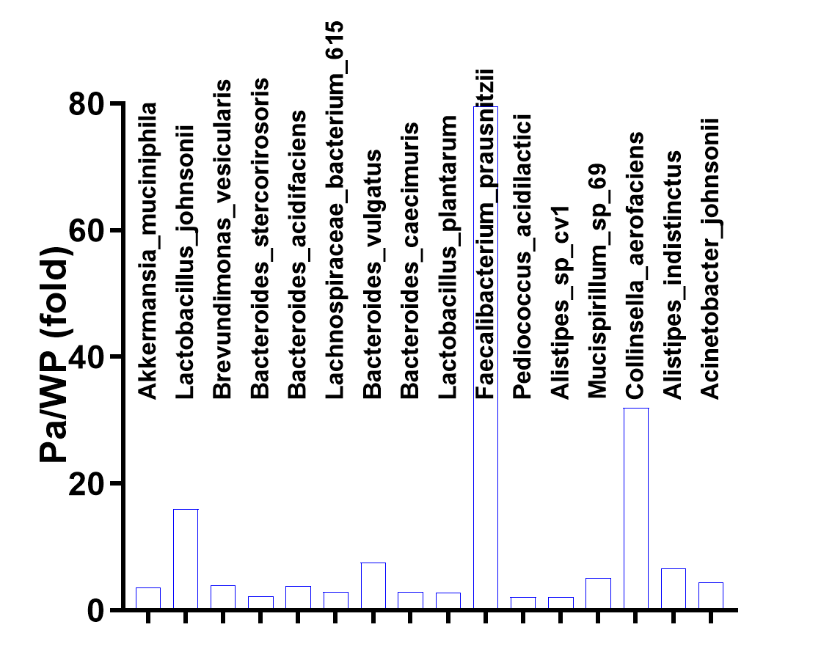


**Fig. S3.** Changes between Pa and WP groups at level of species.

**Figure S4**

**
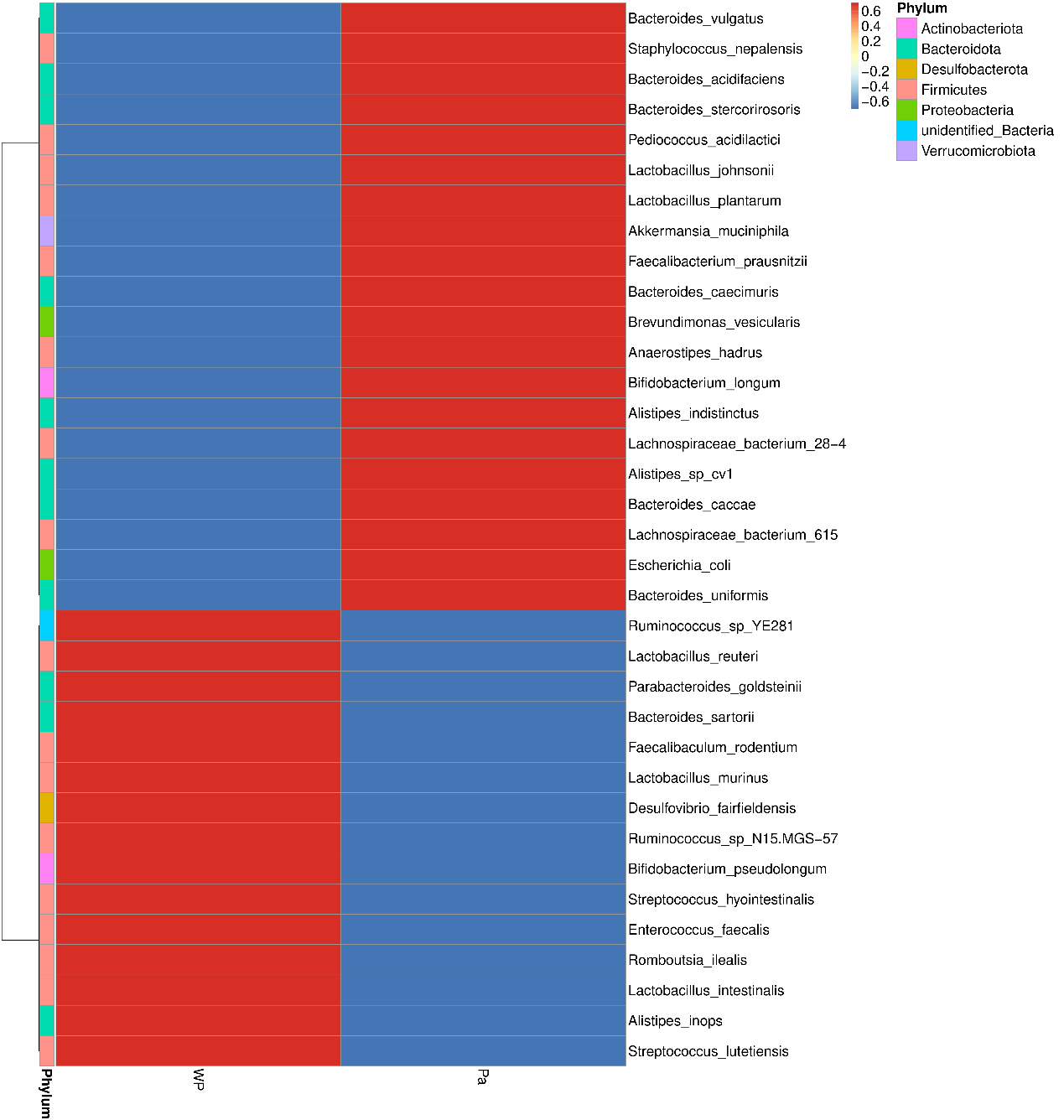
**

**Fig. S4**. Species level abundance heatmaps in WP and Pa groups. Only top 40 dominant species were shown.

**Table S1** The dosage of digested gluten and probiotics used in the mouse model

| Groups | Sensitization (μg) | | | | Challenge (μg) | *P. acidilactici XZ31* (CFU/mL; μL) |
| --- | --- | --- | --- | --- | --- | --- |
|  | 1 | 2 | 3 | 4 | 5  0  0  1200  600  600 |  |
| Ctrl | 0 | 0 | 0 | 0 | 0 | / |
| Ad | 0 | 0 | 0 | 0 | 0 | / |
| WP | 100 | 100 | 100 | 100 | 600 | / |
| Pa | 100 | 100 | 100 | 100 | 600 | Bi: 2.5×10^9^-3×10^9^; 200 |

**Table S2** Quality control of reads preprocessing

| Sample Name | Raw PE(#) | Combined(#) | Qualified(#) | Nochime(#) | Base(nt) | AvgLen(nt) | Q20 | Q30 | GC% | Effective% |
| --- | --- | --- | --- | --- | --- | --- | --- | --- | --- | --- |
| Ad1 | 91,805 | 91,022 | 90,210 | 69,526 | 29396961 | 423 | 98.78 | 95.73 | 54.71 | 75.73 |
| Ad2 | 77,355 | 77,316 | 77,124 | 60,719 | 25257171 | 416 | 98.93 | 96.15 | 53.31 | 78.49 |
| Ad3 | 85,824 | 85,445 | 84,882 | 66,696 | 27548196 | 413 | 98.96 | 96.24 | 53.54 | 77.71 |
| Ad4 | 83,222 | 83,221 | 82,822 | 66,094 | 27947789 | 423 | 98.83 | 95.85 | 54.82 | 79.42 |
| Ad5 | 82,628 | 82,592 | 82,335 | 63,383 | 26288117 | 415 | 98.9 | 96.14 | 54.22 | 76.71 |
| C1 | 80,328 | 79,700 | 79,109 | 60,134 | 25261515 | 420 | 98.81 | 95.83 | 54.47 | 74.86 |
| C2 | 72,489 | 71,905 | 71,389 | 53,960 | 22532114 | 418 | 98.83 | 95.95 | 53.22 | 74.44 |
| C3 | 70,498 | 69,981 | 69,469 | 54,330 | 22247806 | 409 | 98.52 | 95.09 | 54.32 | 77.07 |
| C5 | 82,072 | 81,806 | 81,363 | 64,054 | 26457059 | 413 | 98.75 | 95.83 | 54.56 | 78.05 |
| C6 | 90,452 | 90,061 | 89,436 | 69,707 | 28995539 | 416 | 98.9 | 96.04 | 53.41 | 77.07 |
| P5 | 78,838 | 78,813 | 78,254 | 60,768 | 25386006 | 418 | 98.82 | 95.87 | 52.73 | 77.08 |
| P6 | 80,152 | 79,415 | 78,689 | 61,794 | 25616813 | 415 | 98.88 | 96.04 | 53.49 | 77.1 |
| P7 | 80,887 | 80,800 | 80,406 | 63,281 | 26358339 | 417 | 98.88 | 96.03 | 53.09 | 78.23 |
| P8 | 87,373 | 86,976 | 86,375 | 65,971 | 27435161 | 416 | 98.86 | 95.89 | 53.7 | 75.51 |
| P1 | 88,507 | 88,032 | 87,329 | 67,680 | 28107549 | 415 | 99.03 | 96.43 | 53.53 | 76.47 |
| WP4 | 89,747 | 89,297 | 88,598 | 68,132 | 28462531 | 418 | 98.92 | 96.09 | 54.22 | 75.92 |
| WP5 | 86,906 | 86,380 | 85,824 | 66,654 | 27998766 | 420 | 98.83 | 95.94 | 54.79 | 76.7 |
| WP6 | 77,738 | 77,096 | 76,317 | 58,374 | 24325690 | 417 | 98.71 | 95.7 | 53.86 | 75.09 |
| WP7 | 88,427 | 87,860 | 87,090 | 66,332 | 27768658 | 419 | 98.65 | 95.4 | 54.51 | 75.01 |
| WP8 | 89,289 | 88,662 | 87,711 | 68,785 | 28902229 | 420 | 98.57 | 95.4 | 55.14 | 77.04 |
